# Nasal Microbiota in RSV Bronchiolitis

**DOI:** 10.3390/microorganisms8050731

**Published:** 2020-05-13

**Authors:** Serena Schippa, Antonella Frassanito, Massimiliano Marazzato, Raffaella Nenna, Laura Petrarca, Bruna Neroni, Giulia Bonfiglio, Francesca Guerrieri, Federica Frasca, Giuseppe Oliveto, Alessandra Pierangeli, Fabio Midulla

**Affiliations:** 1 Department of Public Health and Infectious Diseases, “Sapienza” University of Rome, Piazzale Aldo Moro 5, 00185 Rome, Italy; massimiliano.marazzato@uniroma1.it (M.M.); bruna.neroni@uniroma1.it (B.N.); Giulia.bonfiglio@uniroma1.it (G.B.); 2 Department of Maternal, Infantile and Urological Sciences, “Sapienza” University of Rome, Viale Regina Elena 324, 00161 Rome, Italy; raffaella.nenna@uniroma1.it (R.N.); laurapetrarca85@gmail.com (L.P.); 3 Cancer Research Center of Lyon (CRCL), UMR Inserm U1052/CNRS 5286, 69008 Lyon, France; fraguerrieri@gmail.com; 4 Virology Laboratory, Department of Molecular Medicine, “Sapienza” University of Rome, Viale Regina Elena 324, 00161 Rome, Italy; federica.frasca@uniroma1.it (F.F.); giuseppe.oliveto@uniroma1.it (G.O.)

**Keywords:** bronchiolitis, microbiota, respiratory syncytial virus, biodiversity, discriminant species, taxa abundance, microbial networks analyses, microbe interactions

## Abstract

Respiratory Syncytial Virus (RSV) is the leading cause of bronchiolitis, and the severity may be influenced by the bacterial ecosystem. Our aim was to analyze the nasal microbiota from 48 infants affected by bronchiolitis from RSV virus and 28 infants with bronchiolitis but negative for the virus. Results showed a significantly lower biodiversity in the RSV-positive group with respect to the RSV-negative group, a specific microbial profile associated with the RSV-positive group different from that observed in the negative group, and significant modifications in the relative abundance of taxa in the RSV-positive group, as well as in the RSV-A group, with respect to the negative group. Furthermore, microbial network analyses evidenced, in all studied groups, the presence of two predominant sub-networks characterized by peculiar inter- and intra-group correlation patterns as well as a general loss of connectivity among microbes in the RSV-positive group, particularly in the RSV-A group. Our results indicated that infants with more severe bronchiolitis disease, caused by RSV-A infection, present significant perturbations of both the nasal microbiota structure and the microbial relationships. Patients with a milder bronchiolitis course (RSV-B-infected and patients who have cleared the virus) presented less severe alterations.

## 1. Introduction

Bronchiolitis is the most frequent acute respiratory viral infection in children less than one year of age, and it is the main cause of hospitalization in this age group. A viral etiology is detected in 80% of the patients, and Respiratory Syncytial Virus (RSV) remains the most common etiological agent [1]. Several studies have shown that bronchiolitis can be caused also by other respiratory viruses different from RSV, and in some cases a virus cannot be detected. Clinical characteristics of the disease may also be influenced not only by the presence of a specific virus but also by RSV genotype [2]. 

Recent studies have shown that the composition of the nasopharyngeal microbiome may influence the different clinical phenotypes of bronchiolitis [3,4]. In infants with bronchiolitis, the respiratory tract microbiota undergoes a perturbation; several studies showed that the most important qualitative alteration is represented by the increase of Proteobacteria and the decrease of Bacteroidetes [5]. Other studies underline the role of Staphylococcus-dominant airway microbiota during early infancy as “risky microbiota” to enhance viral pathogenesis [6]. A recent study showed that Rhinovirus significantly increased the invasive ability of *Staphylococcus aureus* [7]. Rosas et al. highlight that nasopharyngeal detection and increased abundance of the genus Lactobacillus during RSV infection in infancy is associated with a reduced risk of childhood wheezing illnesses at age 2 years [8]. In 2016, a study from Hasegawa et al. [9] conducted with 40 infants (median age 3 months) hospitalized with bronchiolitis identified four distinct fecal microbiota types in infants. The Bacteroides-dominant profile was associated with a higher probability of bronchiolitis, while the *Enterobacter-*/*Veillonella*-dominant profile was associated with lower possibility of bronchiolitis. Another important recent study, connecting bronchiolitis severity with nasal microbiota composition [10], highlighted four differently dominated nasopharyngeal microbiota profiles: one dominated by *Haemophilus*, another by *Moraxella*, another by *Streptococcus*, and the fourth one showed the highest bacterial richness. Infants in intensive care showed principally *Haemophilus*-dominant profiles. A more recent study, aimed to comprehend the influence of interfaces between the host transcriptome nasal microbiome and the clinical outcomes of RSV infection [11], indicated that specific microbial taxa affect host immune responses. All these studies linked specific bacterial species with RSV bronchiolitis, but no comparisons were carried out between RSV-positive and virus-negative bronchiolitis patients. Understanding the differences of nasal microbiota structures between RSV-positive and virus-negative bronchiolitis patients would give us information on RSV impact/interfere on nasal microbiota structure, and this useful information for new treatments aimed at microbiota rebuilding could promote a non-severe outcome of the disease. In our study, we analyzed nasal microbiota from a total of 76 subjects affected by bronchiolitis, of which 48 were positive for the RSV virus and 28 were negative, in order to explore the nasal microbiota composition. We also analyzed nasal microbiota compositions in infants with bronchiolitis from RSV subtypes A and B.

## 2. Materials and Methods 

### 2.1. Patients and Samples

We conducted a prospective observational study. From October 2016 to May 2017 we enrolled 122 children < 6 months, admitted to the Department of Paediatric Emergency, “Sapienza” University of Rome for an acute episode of bronchiolitis, who had not been on antibiotic therapy within the last 30 days or with probiotics.

Infants with risk factors for severe bronchiolitis (prematurity, congenital heart malformations, chronic lung diseases, and neurological and/or neuromuscular diseases) were excluded.

Bronchiolitis was clinically defined as the first episode of acute lower respiratory tract infection, characterized by the acute onset of cough, tachypnea, retraction, and diffuse crackles on chest auscultation in infants younger than 12 months [12,13]. 

Epidemiological and demographic variables such as gender, age, gestational age, birth weight, delivery, breastfeeding history, presence of siblings, and presence of smoking cohabitants were obtained from the infant’s parents with a structured questionnaire. The following clinical and laboratory data were taken from patient medical files: days of hospitalization, heart rate, respiratory rate, arterial oxygen saturation in room air, presence of retractions, O_2_ therapy, intravenous fluid (IV) therapy, white blood cell count (WBC), lymphocyte count, and C-reactive protein blood concentration (CRP). A clinical severity score ranging from 0 to 8, according to respiratory rate, arterial oxygen saturation in room air, presence of retractions, and IV fluid treatment, was assigned to all infants at admission and every day during hospitalization as previously described [12]. The worst score during hospitalization was used for analysis. Written informed consent was obtained from parents. The study was conducted in accordance with the Declaration of Helsinki and it was approved by institutional review boards of the hospitals (RIF.CE 5271, n. prot. 240/19, 12 March 2019).

Two nasopharyngeal washes (NPWs) were obtained during the first day of hospitalization instilling 3 mL of sterile saline in each nostril and collected with a syringe [12] (the lavages of one nostril were used for virus detection, while the lavage performed on the other nostril was employed for microbiota studies). NPW samples were delivered within 2 h to the Virology Laboratory for virus detection and to the department of Public Health and Infectious Diseases for analysis of the nasal microbiota composition.

### 2.2. Virus Detection and RSV Subtyping

An aliquot of nasopharyngeal washes was used for nucleic acid extraction using a total nucleic acid isolation kit (Roche Diagnostics, Mannheim, Germany). RT-PCR was used to detect the 14 respiratory viruses: Influenza A and B viruses (IV-A/B), human Coronavirus (hCoV) 0C43 229E, NL-63, HUK1, Adenovirus (AV), Parainfluenza virus 1-3 (PIV 1-3), human Metapneumovirus (hMPV), human Bocavirus (hBoV), Respiratory Syncytial Virus (RSV), and Rhinovirus (hRV) [12].

### 2.3. Study of Nasal Microbiota

The NPWs collected were stored at −20 °C or immediately transported to the microbiology lab for nasal microbiota characterization. Total DNA was extracted from all collect nasal samples following the instructions provided by the Kit DNeasy Blood & Tissue (QIAGEN, Hilden, Germany) starting from 200 µL of nasal wash sediment, obtained by centrifuging NPWs at 14,000 rpm and discarded supernatant. To ensure maximum DNA extraction efficiency, even from Gram-positive bacteria, the protocol was modified by adding the incubation of samples with proteinase K at 56 °C, followed by a 4 h incubation at 37 °C with 20 mg/mL (final concentration) of lysozyme (Sigma-Aldrich, Milan, Italy). NanoDrop Spectrophotometer determined the DNA concentration. All DNA samples obtained were normalized to a final DNA concentration of 5 ng/µL in a final volume of 20 µL, and an aliquot was used to characterize nasal bacterial community via next-generation sequencing (NGS) on an Illumina MiSeq platform at the Italian Institute of Technology. To this purpose, the V3–V4 region of the bacterial 16S rRNA gene was amplified by PCR with specific universal bacterial barcoded primer using total DNA extracted as template, and the library obtained was sequenced. The Italian Institute of Technology (IIT) executed the amplicon sequencing by means of next-generation sequencing (NGS) on an Illumina MiSeq platform. Bioinformatic analyses, encompassing metagenomic analysis (multivariate statistics/non-parametric tests and ecological indices analysis), in order to quantify the significant differences in the structure of bacterial communities and cross-correlation of all collected data, were carried out at the Department of Public Health and Infectious Diseases.

Briefly, raw FASTQ files were analyzed with Mothur pipeline v. 1.39.5 for quality check and filtering (sequencing errors, chimerae) on a Workstation DELL T7910 (Round Rock, TX, USA). Raw reads (on average >100k per sample) were filtered (on average >50k per sample) and clustered into Operational Taxonomic Units (OTUs), followed by elimination of low-populated OTUs (till 5 reads) and by de novo OTU picking at 97% pair-wise identity using standardized parameters and the SILVA rDNA Database v. 1.19 for alignment. Sample coverage was computed with Mothur and resulted to be, on average, higher than 99% for all samples, thus meaning a suitable normalization procedure for subsequent analyses. Bioinformatic and statistical analyses on recognized OTUs were performed with Python v. 2.7.11. The most representative and abundant read within each OTU (as evidenced in the previous step with Mothur v. 1.39.5) underwent a nucleotide Blast using the National Center for Biotechnology Information (NCBI) Blast software (ncbi-blast-2.3.0) and the latest NCBI 16S Microbial Database accessed. A matrix of bacterial relative abundances was built at each taxon level (phylum, class, order, family, genus, species) for subsequent multivariate statistical analyses. α-diversity (within sample diversity) was evaluated, in Qiime2 [14], by computing the Shannon index, the Simpson index and the number of observed OTUs at the species level. The analysis of β-diversity (between-sample diversity) was performed by calculating both the Bray–Curtis dissimilarity and the weighted-UniFrac distance. Principal Coordinate Analysis (PCoA) was performed to compare the overall composition of the bacterial community between samples. The differential abundance analysis of taxa was performed at phylum, genus, and species levels by univariate statistical methods (see statistical analysis). A co-occurrence network was computed, at species level, for each group separately (virus negative, RSV positive, RSV-A and RSV-B), by using the SparCC algorithm between abundance of taxa [15]. Only taxa showing a mean relative abundance ≥0.5% in almost one group were included in the network analysis, while SparCC correlations less than −0.5 and greater 0.5 with a *p* ≤ 0.05 based on bootstrapping of 1000 repetitions were retained. Generated networks were imported and visualized in Cytoscape v. 3.7.2. Topological parameters of networks were calculated by using the Network Analyzer plugin included in Cytoscape. 

### 2.4. Statistical Analysis

Firstly, a descriptive analysis of the samples was performed with tables and graphs corresponding to the type of qualitative or quantitative variables. Statistical analysis was carried out using both parametric and nonparametric tests according to the analyzed variable. In particular, non-parametric univariate statistical tests, X_2_ and Fisher’s exact post-hoc tests, were used for the discrete variables; non parametric Mann–Whitney U test and Kruskal–Wallis tests followed by Dunn’s post hoc test were performed to determine significant differences with respect to continuous variables for pairwise or multiple comparisons, respectively. In each case a *p*-value ≤ 0.05 was considered statistically significant. When necessary, the *p* values were corrected by using the Benjamin–Hochberg procedure to account for multiple hypothesis testing. Statistical analyses were performed using SPSS version 25.0 (SPSS Inc., Chicago, IL, USA). 

## 3. Results

### 3.1. Virus Detection

Infants hospitalized with bronchiolitis were tested for respiratory viruses during the winter season 2016/2017. The main clinical characteristics of the studied population are provided in Table 1. Out of 122 NPW samples taken for nasal microbiota characterization, 67 were positive for RSV, and 28 resulted negative to the 14 respiratory viruses tested (V_neg_); 27 samples positive for other respiratory viruses or co-infected were excluded to avoid bias in the analysis. After quality assessment of NGS reads (see below), the study analyses included 28 virus-negative patients and 48 patients infected with RSV. The 48 samples positive only to RSV (RSV_pos_) were sequenced for subtyping as described [2]; of these, 30 were RSV A, 17 were RSV B, and 1 could not be subtyped.

### 3.2. Clinical Variables

The 76 children included in our study were 38 males (50.0%) and had a mean age of 72.3 days (range 23–171). Significantly lower severity scores were observed in the V_neg_ group with respect to the RSV_pos_ (*p* = 0.016). Consistently, the percentage of infants who received O_2_ treatment and were admitted in the pediatric intensive care unit was significantly lower in the virus-negative group compared to either RSV_pos_ groups (respectively *p* = 0.012 and *p* = 0.018). The absolute number of blood lymphocytes was significantly higher in virus-negative infants with respect to the RSV-positive infants (Table 1).

For the other clinical variables, no significant differences were found between the V_neg_ group compared to the RSV_pos_.

### 3.3. Nasal Microbiota Characterization

**Figure 1:**
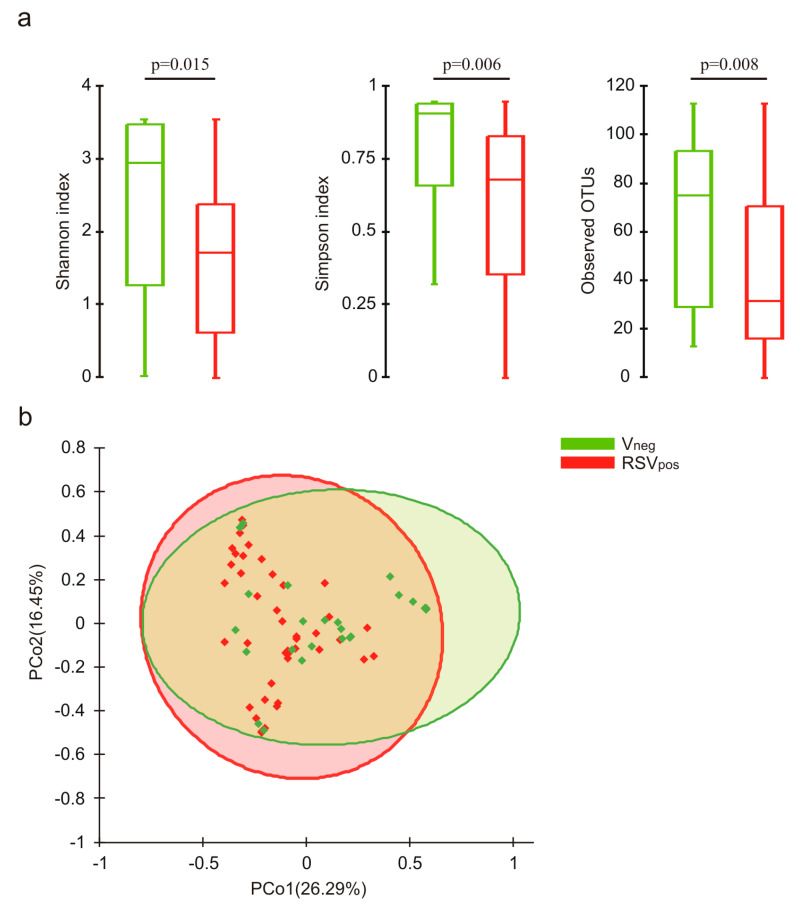
Analysis of microbial biodiversity. (**a**) Evaluation of Shannon index, Simpson index, and number of observed OTUs among groups. *P* values assessing statistical significance were also reported. (**b**) PCoA analysis performed for β diversity based on the Bray–Curtis measure of dissimilarity. For each principal coordinate, the percentage of variance explained is reported between parentheses.

Evaluation of α diversity by the Shannon and Simpson indexes, as well as the number of observed OTUs, showed a significant lower biodiversity in the RSV-positive group with respect to the virus-negative one, suggesting the presence of a microbial shifts in the nasal microbiota of RSV-positive subjects (Figure 1a). A statistically significant difference was found between the RSV-A and the virus-negative group (Shannon *p* = 0.035, Simpson *p* = 0.016, observed OTU *p* = 0.007). No differences were observed between RSV-A and RSV-B groups.

The β diversity analysis showed statistically significant separations between the virus-negative group and both the RSV-positive (*p* = 0.012) and the RSV-A (*p* = 0.010) groups (Figure 1b). No significant partitions were observed between infants negative for the virus and RSV-B groups.

**Figure 2:**
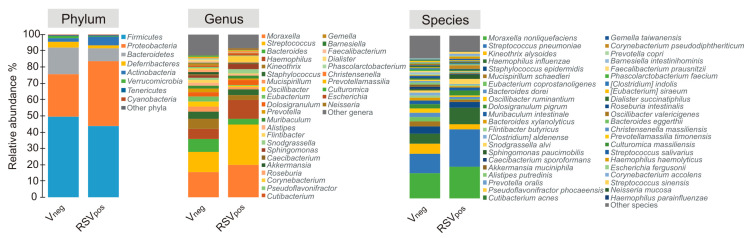
Color-coded bar plot showing the average distribution of bacterial taxa at phylum, genus, and species level across different groups. Only taxa for which a mean relative abundance ≥0.5% was determined in at least one group are shown. Taxa are sorted in ascending order with respect to their mean relative abundance in the V_neg_ group.

Considering only taxa having a mean relative abundance ≥0.5% in at least one of the studied groups, we generated microbial profiles at taxonomy levels of phylum, genera, and species (Figure 2). As shown, distinctive microbial profiles were associated to the different groups (V_neg_, RSV_pos_), but no significant differences in the relative abundance of taxa were observed at phylum level among all groups.

**Figure 3:**
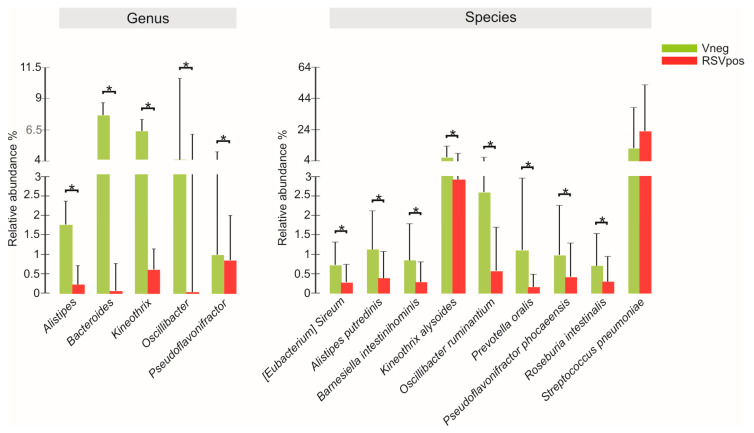
Color-coded bar plot showing differential abundance analysis at genus and species levels performed by Mann–Whitney U tests. * p value ≤ 0.05.

The analysis of the relative abundance of the genera among the different groups (Figure 3) showed that Bacteroides, Pseudoflavonifractor, Alistipes, Kineothrix, and Oscillibacter were significantly lower in abundance in the RSV_pos_ group compared to the V_neg_. At species level, the relative abundance of *Streptococcus pneumoniae* was significantly higher in the RSV_pos_ group. The same statistically significant differences observed between RSV_pos_ and V_neg_ groups were kept when we matched the RSV-A and the V_neg_ groups. No significant differences were highlighted between V_neg_ and RSV-B groups nor between the RSV-A and RSV-B ones, although we cannot exclude that the lack of statistical significance was due to the limited number of infants positive for RSV-B.

### 3.4. Network Analysis

**Figure 4:**
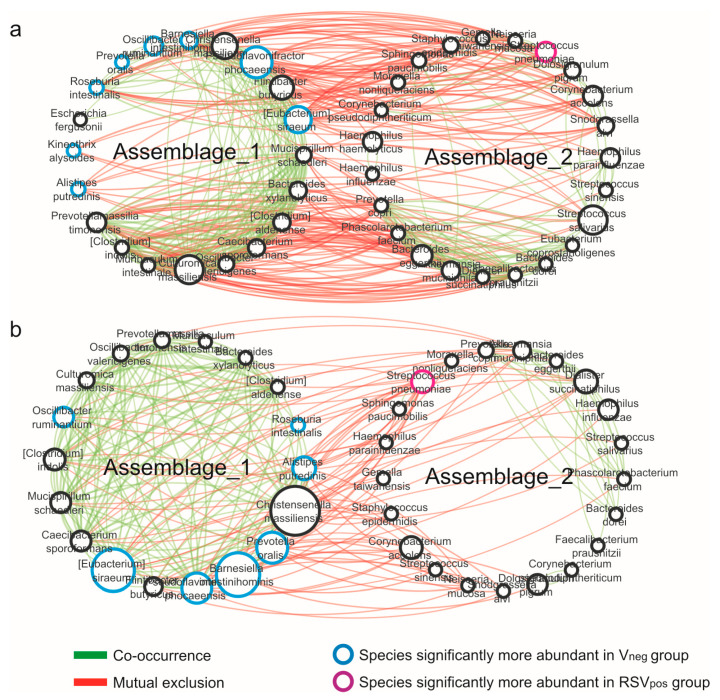
Co-occurrence network analysis. Graphical representations of (**a**) virus-negative and (**b**) RSV_pos_ networks, computed at species level performed on taxa with a mean relative abundance ≥0.5% in at least one group. The thickness of edges represents the level of association between taxa based on the SparCC score. The size of nodes is proportional to the number of edges departing from the node, indicating its degree of interaction.

Interactions between taxa were explored by performing a network analysis based on the SparCC algorithm, which is able to reduce bias introduced by the compositionality and sparsity of microbiome data. The graphical representation of the microbial networks constitutes a sort of “correlation map” describing the positive and negative relationships between microbes drawn as edges with different colors. In this context, positive and negative correlations could reflect synergistic and antagonistic interactions between microbial groups such as metabolic interdependencies or competitions for the same ecological niches. Results evidenced the presence of two predominant microbial assemblages (named assemblage 1 and 2) in analyzed networks (V_neg_, RSV_pos_), in which taxa correlated with synergic interactions within the assemblage. Differently competitive interactions between the taxa belonging to the two different assemblages were observed (Figure 4). Remarkable are the taxa compositions of the two assemblages. In both cases, assemblage 1 was characterized by bacterial species resulting to be significantly more abundant within the virus-negative group, while *S. pneumoniae,* the unique species significantly associated with the RSV_pos_ group, was within assemblage 2.

The co-occurrence networks were evaluated for topological properties including centrality measures (Table 2). Obtained results showed a decrease in the number of nodes (taxa participating to the network) in RSV_pos._ Similarly, results were obtained for the number of correlations (edges) indicating a network with less connection among bacteria in RSV_pos_ with respect to the network determined for the V_neg_ group. Concerning correlation type, a decrease in both positive/synergic and negative/competitive was found in the RSV_pos_ network with respect to V_neg_; however, this could be due to the overall lower number of connections present in the RSV_pos_ network. The decrease of relationships among bacterial species strongly evidenced that changes happened in the nasal microbial ecosystem structure, leading to an impoverished microbial network characterized by weaker interactions among microbes, a reduced interconnection among species (Figure 4), and the loss of reciprocal control between microbes. These characteristics depict a condition in which the eubiotic state has been lost, determining a state of dysbiosis.

## 4. Discussion

The main aim of the present study was to characterize the nasal microbiota in pediatric patients hospitalized for bronchiolitis from RSV and in infants affected by bronchiolitis but negative for a respiratory virus. Furthermore, we attempted to characterize the nasal microbiota in infants with bronchiolitis from RSV-A and patients with bronchiolitis from RSV-B.

Nasal microbiota characterization was firstly carried out by evaluating the ecological indexes (α diversity). The ecological perception is fundamental to better understand the dynamic of the microbial ecosystem, and it is important to develop therapeutic approaches aimed to rebuild a balanced microbiota [16]. Subsequently, we evaluated the β diversity to exploit the differences in the nasal microbial composition among the groups studied. We highlighted significant differences among the groups; in particular, we showed the following: 

(i) a significantly higher biodiversity in the virus-negative group with respect to the RSV-positive and to the RSV-A groups;

(ii) a specific nasal microbiota composition in the virus-negative group, different from that of the RSV-positive and RSV-A groups; and

(iii) a significant modification in taxa relative abundance, among RSV-positive, in particular the RSV-A, with respect to the virus-negative group. 

Several of the bacterial species less abundant in the virus-positive group are known to be potentially beneficial species such as butyrate producers, species proposed as probiotic, or anti-inflammatory species [17,18,19]. The infants were negative to PCR-based tests for 14 respiratory viruses. They probably experienced a viral infection causing acute respiratory symptoms, but may have tested negative at the time of hospitalization due to viral load decline. Concomitantly, they had a less severe clinical course of bronchiolitis. It can be considered a group in which the viral clearance of the upper airways is actually more efficient and faster (this could derive from a more resistant environment or from a more efficient immune response), or it could be a group of patients in which we were unable to isolate the virus because it is not part of our identification panel for the 14 respiratory viruses. Interestingly, these infants’ nasal microbiota are characterized by a relatively high alpha diversity, by the presence of potentially beneficial species, and by more connections in their microbial ecosystem that could be associated to a milder clinical course. On the other hand, a higher severity was observed in the RSV-A-infected group, characterized by the abundance of the unique species *S. pneumoniae* that is associated with several diseases such as pneumonia, otitis, and meningitis, but that can be part of the respiratory microbiota without causing any pathology. Our results indicate that in RSV-A-positive group, the relative abundance of *S. pneumoniae* was significantly higher than that in the virus-negative group. These data support the clinical evidence that *S. pneumoniae* colonization is associated with increased severity during RSV infection in young children [20]. The presence of *S. pneumoniae* in our RSV-negative group is easily explained by the fact that our virus-negative group was affected by bronchiolitis too. Furthermore, *S. pneumoniae* should be considered as a pathobiont bacterial species, meaning a potentially pathogenic species present in low quantity in healthy subjects that, in specific conditions under a specific selective pressure (maybe virus presence), could become predominant by establishing a dysbiosis status in the ecosystem. It has been reported that *S. pneumoniae* colonization and its density can enhance the risk of severed subsequent RSV infection [21], but this association may have bidirectional interactions. RSV-A somehow promotes *S. pneumoniae* growth, directly [22] or through the inflammatory response induced by the viral infection, as shown for influenza virus [23,24]. As reported in the literature for influenza virus co-infections, the nasal microbiota composition could affect individual predisposition to secondary bacterial infections, commonly triggered by Gram-positive bacteria like *S. pneumoniae* [25], and has a direct impact on pneumococcal attitude [26]. Moreover, in host infected with influenza virus, the pro-inflammatory immune response represents a key aspect relevant to lethal pneumococcal infection, and it denotes a potential target for therapeutic intervention [23,27]. Our results corroborate results by other authors indicating the presence of *Streptococcus* species associated with RSV bronchiolitis and its severity. A study from de Steenhuijsen Piters et al. [28] aimed to evaluate, in infants less than 2 years of age, if definite nasopharyngeal microbiota were associated with different host transcriptome profiles. They found RSV infection severity related to five nasopharyngeal microbiota groups, categorized by the enhancement of a specific bacterial species. Children with RSV infection were associated with a microbiota dominated by *H. influenzae* and *Streptococcus*. Furthermore, the study showed that communications among RSV and nasopharyngeal microbiota might regulate the host immune response, hypothetically affecting clinical disease severity. Another recent study [29] pointed out that *Streptococcus* abundance was greater in samples collected during RSV infection compared with samples collected one month later.

Finally, we evaluated the relations among microbial community members by networks analysis, as edges that join pairs of nodes (the system components, taxa). Microbial networks analyses evidenced two predominant sub-networks in all groups studied (virus negative, RSV positive); it would seem that the two sub-networks control each other. Interestingly, the majority of taxa resulted to be significantly more abundant in the virus-negative group and were placed in sub-network 1, while the unique species significantly more abundant in the RSV_pos_ group, *S. pneumoniae*, was in sub-network 2 and, therefore, in negative/competitive correlation with the others in both groups studied. Despite the overall similarity of the two sub-networks found, we observed variations among groups in taxa interactions. A microbial ecosystem with fewer connections among microbes, and probably more susceptible to colonization by outdoor microbes, was observed in the RSV-positive group with respect to the virus-negative group. Several taxa in the RSV-positive group are no longer connected and present as isolated elements. The lower connection between microbes could indicate dysbiosis, in which the mutualistic relationships among taxa are lost. 

Enrolled infants were between 3 weeks and 6 months of age and experiencing their first severe acute respiratory infection that caused hospitalization. During this period of our life nasal microbiota composition is still very mutable, in particular in the second and third months of age [30]. The first colonizing microbes have a strong influence on what will be the following colonizers, through the expression modulation of genes coding for adhesion sites [31,32].

Notwithstanding, we found interesting differences in microbiota composition in infants when comparing, on the basis of RSV infection, groups that were similar for mean age, weight, and type of feeding, and differed for bronchiolitis severity. This is the first study that pointed out differences in nasal microbiota composition among RSV-positive and virus-negative bronchiolitis patients, and it denoted, for the first time, a more robust impact on nasal microbiota structure seemed to be related to RSV subtype A compared to RSV subtype B. Although no significant differences were determined between the RVS-B group and the V_neg_ or the RSV-A groups, we cannot exclude that the lack of statistical significance could be due to the limited number of analyzed RSV-B subjects, which represents a clear limit for this study. Undoubtedly, the presence of RSV represents a disturbing factor during the first colonization phase of the nasal site, which could profile microbiota not in eubiotic equilibrium, increasing the risk of illnesses caused by microbes whose growth is favored by RSV (e.g., *S. pneumoniae*) as well as the risk of respiratory sequelae [33,34]. Differently, a resilient respiratory microbiota may favor a decrease in viral load and a less severe illness course.

## 5. Conclusions

In conclusion, our results showed that two specific sub-clusters are present in the networks of the nasal microbiota in all our bronchiolitis patients, independently from RSV presence. Moreover, this analysis highlighted, for the first time, a network in the RSV_pos_ group where several taxa species have lost interaction with others and remain as bacterial species apparently disconnected from the ecosystem, shaping a state of dysbiosis (Figure 4). Accordingly, the infants affected by RSV bronchiolitis presented more perturbations in the nasal microbiota structure compared to the V_neg_ group, thus favoring colonization of pathobiont species, such as *S. pneumonia*, and negatively influencing potentially beneficial species growth, defining a state of dysbiosis. All of this goes with the higher disease severity score, indicating that a dysbiosis status emphasized by RSV presence could itself act as a predisposing factor for bronchiolitis severity.

Longitudinal study approaches are necessary to clarify whether the observed associations are causally linked and whether the control of pathobionts overgrowth, by therapies aimed at improving the nasal microbiota composition, may contribute to prevent a severe bronchiolitis course.

## Figures and Tables

**Table 1:** Clinical characteristics of the studied population. Qualitative clinical variables are reported as number of occurrences and percentage, while continuous variables are expressed as mean ± SD. The reported *p* values are relative to chi square tests or Mann–Whitney U tests performed between the V_neg_ group and the RSV_pos_ group. *p* < 0.05 was considered statistically significant.

Characteristics	V_neg_ (*N* = 28)	RSV_pos_ (*N* = 48)	*p* Value
Male	13 (46.4%)	25 (52.1%)	0.634
Caesarean section	12 (42.9%)	27 (43.8%)	0.940
Age (days)	65.7 ± 3 4.8	76.3 ± 34.5	0.139
Weight	5.0 ± 1.2	5.3 ± 1.0	0.291
Hospitalization (days)	3.7 ± 1.6	4.8 ± 2.4	0.071
Severity score	3.0 ± 1.6	3.9 ± 1.5	0.016
O_2_ therapy	3 (10.7%)	18 (37.5%)	0.012
Admission to PICU	0 (0.0%)	3 (6.3%)	0.018
White blood cells	11083.2 ± 3615.9	9784.4 ± 2965.8	0.189
Lymphocytes absolute	5683.2 ± 1689.1	4797.4 ± 2039.4	0.030
Neutrophils absolute	3323.7 ± 2464.9	3166.5 ± 1967.3	0.834
Eosinophil absolute	212.7 ± 192.9	136.7 ± 190.5	0.062
Eosinophil > 400	4 (14.3%)	2 (4.2%)	0.115
Breastfeeding	15 (55.61%)	31 (64.6%)	0.441

**Table 2:** Topological properties of networks.

Properties	V_neg_	RSV_pos_
Nodes	43	39
Edges	221	124
Edges/node ratio	5.14	3.18
Synergisms	121	85
Exclusions	100	39
Syn/Escl ratio	1.21	2.17
Average number of neighbors	10.28	6.359
Network density	0.245	0.167
